# A one-dimensional copper(II) phenyl­ene­diphospho­nate: *catena*-poly[[(1,10-phenanthroline-κ^2^ *N*,*N*′)copper(II)]-μ_3_-[*m*-phenyl­enediphospho­nato-κ^3^ *O*:*O*′:*O*′′]]

**DOI:** 10.1107/S1600536810037359

**Published:** 2010-09-30

**Authors:** Paul DeBurgomaster, Jon Zubieta

**Affiliations:** a Department of Chemistry, Syracuse University, Syracuse, New York 13244, USA

## Abstract

The title compound, [Cu(1,3-HO_3_PC_6_H_4_PO_3_H)(C_12_H_8_N_2_)]_*n*_, is a coordination polymer of the metal–diphospho­nate family. The chain structure is constructed from ‘4+1’ square-py­rami­dally coordinated copper(II) atoms bonded to chelating phenanthroline (phen) ligands and linked through 1,3-phenyldihydrogendiphospho­nate ligands. The basal plane of the Cu(II) site is defined by the phen nitro­gen donors and phospho­nate oxygen atoms from two diphospho­nate ligands, while the apical position is occupied by an oxygen donor from a third diphospho­nate ligand. The chains propagate along the *a*-axis direction. Inversion-related phen groups engage in π-π stacking with a mean distance of 3.376 (2) Å between the ring planes. O—H⋯O hydrogen-bonding inter­actions between the protonated {P—OH} groups of one chain and the {P=O} groups of adjacent chains stabilize the crystal packing.

## Related literature

For general background to metal-organo­phospho­nates, see: Clearfield (1998 ▶); Finn *et al.* (2003 ▶); Vermeulen (1997 ▶). For copper-organo­phospho­nates, see: DeBurgomaster *et al.* (2010 ▶) and references therein; Arnold *et al.* (2002 ▶) and references therein. For our recent studies of metal-organo­phospho­nates, see: Armatas *et al.* (2009 ▶); Ouellette *et al.* (2009 ▶). For the catalytic, ion exchange, sensor and non-linear optical properties of transition metal compounds of organo­phospho­nic ligands, see: Bakmutova *et al.* (2008 ▶); Konar *et al.* (2007 ▶); Vermeulen (1997 ▶); Turner *et al.* (2003 ▶).
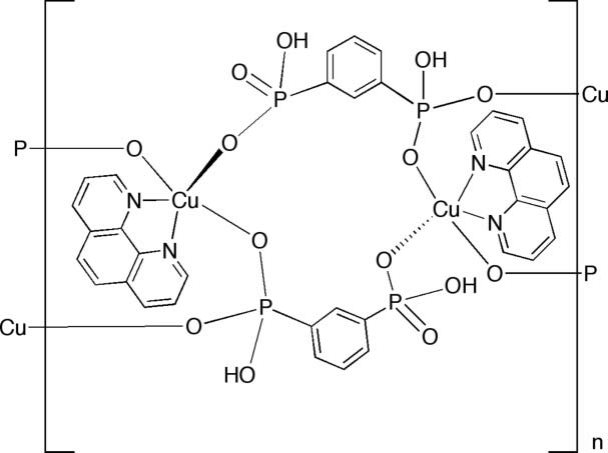


## Experimental

### 

#### Crystal data

[Cu(C_6_H_6_O_6_P_2_)(C_12_H_8_N_2_)]
*M* *_r_* = 479.79
Triclinic, 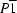

*a* = 8.6142 (10) Å
*b* = 9.0554 (10) Å
*c* = 12.1094 (13) Å
α = 99.688 (2)°
β = 106.542 (2)°
γ = 98.184 (2)°
*V* = 874.30 (17) Å^3^
*Z* = 2
Mo *K*α radiation
μ = 1.48 mm^−1^
*T* = 98 K
0.35 × 0.30 × 0.21 mm

#### Data collection

Bruker APEX CCD area-detector diffractometer
Absorption correction: multi-scan (*SADABS*; Bruker, 1998 ▶) *T*_min_ = 0.626, *T*_max_ = 0.747
8704 measured reflections
4190 independent reflections
4042 reflections with *I* > 2σ(*I*)
*R*_int_ = 0.018

#### Refinement

*R*[*F* ^2^ > 2σ(*F* ^2^)] = 0.033
*wR*(*F* ^2^) = 0.087
*S* = 1.09
4190 reflections
262 parameters
H-atom parameters constrained
Δρ_max_ = 0.71 e Å^−3^
Δρ_min_ = −0.67 e Å^−3^

### 

Data collection: *SMART* (Bruker, 1998 ▶); cell refinement: *SAINT* (Bruker, 1998 ▶); data reduction: *SAINT*; program(s) used to solve structure: *SHELXS97* (Sheldrick, 2008 ▶); program(s) used to refine structure: *SHELXL97* (Sheldrick, 2008 ▶); molecular graphics: *CrystalMaker* (Palmer, 2006 ▶); software used to prepare material for publication: *SHELXTL* (Sheldrick, 2008 ▶).

## Supplementary Material

Crystal structure: contains datablocks I, global. DOI: 10.1107/S1600536810037359/pk2259sup1.cif

Structure factors: contains datablocks I. DOI: 10.1107/S1600536810037359/pk2259Isup2.hkl

Additional supplementary materials:  crystallographic information; 3D view; checkCIF report

## Figures and Tables

**Table 1:** Hydrogen-bond geometry (Å, °)

*D*—H⋯*A*	*D*—H	H⋯*A*	*D*⋯*A*	*D*—H⋯*A*
O2—H2⋯O3^i^	0.84	1.81	2.489 (2)	136
O5—H5⋯O1^ii^	0.84	1.74	2.574 (2)	173

Symmetry codes: (i) 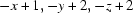; (ii) 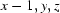.

**Fig. 1.**
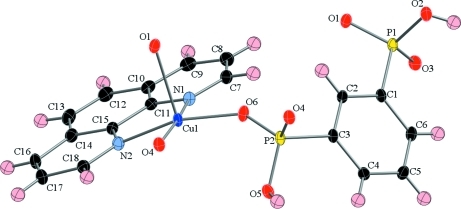
The asymmetric unit of the title compound, showing the atom-labeling scheme and displacement ellipsoids at the 50% probability level. Color scheme: copper, blue; phosphorus, yellow; nitrogen, light blue; oxygen, red; carbon, black; hydrogen, pink.

**Fig. 2.**
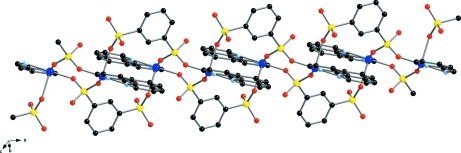
A view of the chain structure of [Cu(phen)(1,3-HO3PC6H4PO3H)]n which propagates along the a-axis direction. The π-π stacking of phen groups is also shown. Color scheme as for Fig. 1.

**Fig. 3.**
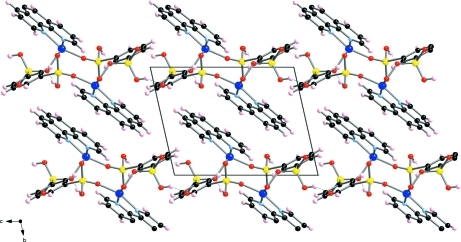
The packing diagram of the compound viewed down the a axis. Hydrogen bonds are shown as dashed lines. Color scheme as for Fig. 1.

## supplementary crystallographic information

### Comment

Metal organophosphonate materials are prototypical organic-inorganic hybrid composites, often exhibiting layered or pillared-layer structures (Clearfield, 1998; Finn *et al.*, (2003)). A variety of transition metal compounds of organophosphonic ligands have been investigated for their catalytic, ion exchange, sensor and non-linear optical properties (Bakmutova *et al.* (2008); Konar *et al.*,(2007); Vermeulen, (1997); Turner *et al.* (2003)). In the specific case of copper-organophosphonate materials, layered structures are the most common, adopting the prototypical 'pillared' layer motif (Arnold, *et al.* (2002)). In the course of our extensive studies of metal-organophosphonate chemistry (Armatas *et al.* (2009); Ouellette *et al.* (2009); DeBurgomaster, *et al.* (2010)), we have noted the structural influences of coligands and/or secondary metal-organic moieties. For the title compound, [Cu(phen)(1,3-HO_3_PC_6_H_4_PO_3_H)] (Fig.), the bidentate phenanthroline ligand by occupying three coordination sites about the Cu(II) centers constrains structural extension to one-dimension. The material exhibits a chain motif running parallel to the [100] direction (Fig. 2). The five coordinate {CuO_3_N_2_} geometry at the Cu(II) site is defined by the nitrogen donors of the chelating phenanthroline ligand and oxygen donors from two distinct 1,3-phenyldiphosphonate ligands in the basal and an oxygen donor from a third diphosphosphonate ligand in the apical position. The '4 + 1' axially distorted Jahn-Teller geometry exhibits an elongated Cu—O bond length of 2.292 (2) Å, compared to Cu—O bond distances of 1.934 (2)Å and 1.937 (2)Å for the oxygen donors in the basal plane. Each phenyldiphosphonate ligand bridges three copper sites in the chain. The resultant connectivity pattern generates two repeating heterocyclic rings; the first consists of two copper sites bridged by two diphosphonate ligands to give the sixteen-membered {–Cu—O—P—C—C—C—P—O–}_2_ ring while the second is the common {M2(µ_2_-phosphonate-O,*O*')_2_} motif or in this case the eight-membered {–Cu—O—P—O–}_2_ ring. The alternating ring structure is similar to that observed for the previously reported [Cu(2,2'-bipyridine)(1,3,5-(HO_3_P)_2_C_6_H_3_PO_3_H_2_)] (DeBurgomaster, *et al.* (2010)). Charge-balance requirements dictate that the diphosphonate ligand must be doubly protonated, that is (HO_3_PC_6_H_4_PO_3_H)^2-^. The P2—O5 bond distance of 1.574 (2) Å, compared to distances of 1.509 (2)Å and 1.517 (2)Å for P2—O4 and P2—O6, establishes O5 as one protonation site, an observation confirmed by the appearance of a peak consistent with the O5 proton on the difference Fourier map. The location of the second proton is less clear with O2 and O3 as possibilities. Based on the appearance of a peak consistent with an O2 proton in the difference Fourier, oxygen atom O2 was deemed the site of protonation. The pendant {P = O} and {P—OH} groups of adjacent chains engage in hydrogen-bonding to link the chains into a three-dimensional framework (Fig. 3).

### Experimental

A mixture of copper acetate monohydrate (0.096 g, 0.48 mmol), 1,10-phenanthroline (0.117 g, 0.50 mmol), 1,3-phenyldiphosphonic acid (0.118 g, 0.50 mmol), and H_2_O (10.00 ml, 554.94 mmol) in the mole ratio 1.00:1.04:1.04:1156 was heated to 170°C for 4 days. Initial and final pH values of 3.0 and 3.0, respectively, were recorded. Blue rods suitable for X-ray diffraction were isolated in 70% yield. Anal. Calcd. for C_18_H_14_CuN_2_O_6_P_2_: C, 45.0; H, 2.92; N, 5.84. Found: C, 44.8; H, 2.86; N, 5.95.

### Refinement

Hydrogen atoms of the phenanthroline ring and the phosphonate protons were located on the difference Fourier and were subsequently positioned geometrically with C—H = 0.95 Å and O—H = 0.84 Å. These latter hydrogen atoms were constrained to ride on their parent atoms with *U*_iso_(H) = 1.2 *x U*_iso_(C) and *U*_iso_(H) = 1.5 *x U*_iso_(O).

### Crystal data

**Table:**

[Cu(C_12_H_8_N_2_)(C_6_H_6_O_6_P_2_)]	*Z* = 2
*M**_r_* = 479.79	*F*(000) = 486
Triclinic, *P*1	*D*_x_ = 1.823 Mg m^−^^3^*D*_m_ = 1.81 (2) Mg m^−^^3^*D*_m_ measured by not measured
Hall symbol: -P 1	Mo *K*α radiation, λ = 0.71073 Å
*a* = 8.6142 (10) Å	Cell parameters from 5367 reflections
*b* = 9.0554 (10) Å	θ = 2.3–28.4°
*c* = 12.1094 (13) Å	µ = 1.48 mm^−^^1^
α = 99.688 (2)°	*T* = 98 K
β = 106.542 (2)°	Block, blue
γ = 98.184 (2)°	0.35 × 0.30 × 0.21 mm
*V* = 874.30 (17) Å^3^	

### Data collection

**Table:**

Bruker APEX CCD area-detector diffractometer	4190 independent reflections
Radiation source: fine-focus sealed tube	4042 reflections with *I* > 2σ(*I*)
graphite	*R*_int_ = 0.018
φ and ω scans	θ_max_ = 28.1°, θ_min_ = 1.8°
Absorption correction: multi-scan (*SADABS*; Bruker, 1998)	*h* = −11→11
*T*_min_ = 0.626, *T*_max_ = 0.747	*k* = −11→11
8704 measured reflections	*l* = −15→15

### Refinement

**Table:**

Refinement on *F*^2^	Primary atom site location: structure-invariant direct methods
Least-squares matrix: full	Secondary atom site location: difference Fourier map
*R*[*F*^2^ > 2σ(*F*^2^)] = 0.033	Hydrogen site location: inferred from neighbouring sites
*wR*(*F*^2^) = 0.087	H-atom parameters constrained
*S* = 1.09	*w* = 1/[σ^2^(*F*_o_^2^) + (0.0425*P*)^2^ + 1.0147*P*] where *P* = (*F*_o_^2^ + 2*F*_c_^2^)/3
4190 reflections	(Δ/σ)_max_ = 0.001
262 parameters	Δρ_max_ = 0.71 e Å^−^^3^
0 restraints	Δρ_min_ = −0.67 e Å^−^^3^

### Special details

**Table:**

Geometry. All esds (except the esd in the dihedral angle between two l.s. planes) are estimated using the full covariance matrix. The cell esds are taken into account individually in the estimation of esds in distances, angles and torsion angles; correlations between esds in cell parameters are only used when they are defined by crystal symmetry. An approximate (isotropic) treatment of cell esds is used for estimating esds involving l.s. planes.
Refinement. Refinement of *F*^2^ against ALL reflections. The weighted *R*-factor *wR* and goodness of fit *S* are based on *F*^2^, conventional *R*-factors *R* are based on *F*, with *F* set to zero for negative *F*^2^. The threshold expression of *F*^2^ > σ(*F*^2^) is used only for calculating *R*-factors(gt) *etc*. and is not relevant to the choice of reflections for refinement. *R*-factors based on *F*^2^ are statistically about twice as large as those based on *F*, and *R*- factors based on ALL data will be even larger.

### Fractional atomic coordinates and isotropic or equivalent isotropic displacement parameters (Å^2^)

**Table:**

	*x*	*y*	*z*	*U*_iso_*/*U*_eq_	
Cu1	0.32660 (3)	0.82971 (3)	0.58802 (2)	0.01190 (8)	
P1	0.32369 (6)	1.03977 (6)	0.85348 (4)	0.01186 (11)	
P2	−0.33649 (6)	1.05190 (6)	0.64714 (4)	0.01144 (11)	
O1	0.31787 (17)	1.03279 (17)	0.72590 (12)	0.0148 (3)	
O2	0.33003 (18)	0.88390 (17)	0.88644 (13)	0.0163 (3)	
H2	0.3720	0.8969	0.9599	0.025*	
O3	0.46333 (18)	1.16671 (18)	0.94061 (13)	0.0162 (3)	
O4	−0.43602 (17)	0.89210 (17)	0.62759 (13)	0.0138 (3)	
O5	−0.44080 (17)	1.17467 (17)	0.67394 (13)	0.0151 (3)	
H5	−0.5181	1.1348	0.6956	0.023*	
O6	−0.26911 (17)	1.08135 (18)	0.54895 (13)	0.0151 (3)	
N1	0.0864 (2)	0.7410 (2)	0.55889 (15)	0.0134 (3)	
N2	0.3570 (2)	0.6720 (2)	0.68729 (15)	0.0138 (3)	
C1	0.1322 (2)	1.0872 (2)	0.86658 (17)	0.0124 (4)	
C2	−0.0105 (2)	1.0504 (2)	0.76856 (17)	0.0130 (4)	
H2A	−0.0062	1.0002	0.6943	0.016*	
C3	−0.1596 (2)	1.0864 (2)	0.77792 (17)	0.0122 (4)	
C4	−0.1667 (2)	1.1571 (2)	0.88791 (18)	0.0145 (4)	
H4	−0.2678	1.1808	0.8953	0.017*	
C5	−0.0249 (3)	1.1930 (2)	0.98692 (18)	0.0160 (4)	
H5A	−0.0298	1.2404	1.0617	0.019*	
C6	0.1235 (2)	1.1592 (2)	0.97584 (18)	0.0143 (4)	
H6	0.2198	1.1853	1.0431	0.017*	
C7	−0.0474 (3)	0.7773 (2)	0.49048 (18)	0.0153 (4)	
H7	−0.0340	0.8447	0.4401	0.018*	
C8	−0.2076 (3)	0.7190 (3)	0.49042 (19)	0.0184 (4)	
H8	−0.3007	0.7471	0.4408	0.022*	
C9	−0.2286 (3)	0.6210 (3)	0.56286 (19)	0.0179 (4)	
H9	−0.3364	0.5812	0.5636	0.021*	
C10	−0.0895 (3)	0.5798 (2)	0.63595 (18)	0.0154 (4)	
C11	0.0661 (2)	0.6437 (2)	0.62948 (18)	0.0134 (4)	
C12	−0.0956 (3)	0.4795 (3)	0.7153 (2)	0.0187 (4)	
H12	−0.1994	0.4363	0.7209	0.022*	
C13	0.0445 (3)	0.4451 (3)	0.78248 (19)	0.0192 (4)	
H13	0.0370	0.3798	0.8353	0.023*	
C14	0.2038 (3)	0.5059 (2)	0.77509 (18)	0.0165 (4)	
C15	0.2133 (3)	0.6059 (2)	0.69964 (18)	0.0138 (4)	
C16	0.3533 (3)	0.4700 (3)	0.83802 (19)	0.0194 (4)	
H16	0.3534	0.4010	0.8891	0.023*	
C17	0.4990 (3)	0.5359 (3)	0.82459 (19)	0.0192 (4)	
H17	0.6005	0.5120	0.8659	0.023*	
C18	0.4971 (3)	0.6387 (2)	0.74959 (18)	0.0166 (4)	
H18	0.5988	0.6860	0.7430	0.020*	

### Atomic displacement parameters (Å^2^)

**Table:**

	*U*^11^	*U*^22^	*U*^33^	*U*^12^	*U*^13^	*U*^23^
Cu1	0.00830 (12)	0.01613 (14)	0.01135 (13)	0.00099 (9)	0.00196 (9)	0.00652 (9)
P1	0.0083 (2)	0.0170 (3)	0.0100 (2)	0.00289 (18)	0.00145 (18)	0.00446 (19)
P2	0.0076 (2)	0.0163 (3)	0.0108 (2)	0.00190 (18)	0.00179 (18)	0.00644 (18)
O1	0.0121 (6)	0.0218 (8)	0.0115 (7)	0.0033 (6)	0.0042 (5)	0.0056 (6)
O2	0.0172 (7)	0.0183 (7)	0.0130 (7)	0.0043 (6)	0.0024 (6)	0.0053 (6)
O3	0.0107 (6)	0.0199 (8)	0.0160 (7)	0.0019 (6)	0.0014 (5)	0.0044 (6)
O4	0.0090 (6)	0.0176 (7)	0.0145 (7)	0.0013 (5)	0.0026 (5)	0.0058 (6)
O5	0.0109 (6)	0.0186 (7)	0.0172 (7)	0.0041 (6)	0.0038 (5)	0.0080 (6)
O6	0.0106 (6)	0.0240 (8)	0.0123 (7)	0.0029 (6)	0.0033 (5)	0.0089 (6)
N1	0.0130 (8)	0.0138 (8)	0.0129 (8)	0.0012 (6)	0.0035 (6)	0.0039 (6)
N2	0.0122 (8)	0.0165 (8)	0.0128 (8)	0.0031 (6)	0.0032 (6)	0.0043 (6)
C1	0.0102 (8)	0.0153 (9)	0.0126 (9)	0.0028 (7)	0.0028 (7)	0.0063 (7)
C2	0.0119 (9)	0.0163 (10)	0.0112 (9)	0.0027 (7)	0.0035 (7)	0.0044 (7)
C3	0.0094 (8)	0.0153 (9)	0.0113 (9)	0.0006 (7)	0.0020 (7)	0.0053 (7)
C4	0.0125 (9)	0.0181 (10)	0.0151 (10)	0.0034 (7)	0.0057 (7)	0.0069 (8)
C5	0.0161 (9)	0.0202 (10)	0.0122 (9)	0.0036 (8)	0.0051 (8)	0.0041 (8)
C6	0.0117 (9)	0.0176 (10)	0.0116 (9)	0.0009 (7)	0.0007 (7)	0.0046 (7)
C7	0.0138 (9)	0.0169 (10)	0.0142 (9)	0.0024 (8)	0.0029 (7)	0.0043 (8)
C8	0.0130 (9)	0.0224 (11)	0.0181 (10)	0.0042 (8)	0.0026 (8)	0.0029 (8)
C9	0.0125 (9)	0.0200 (10)	0.0196 (10)	−0.0002 (8)	0.0063 (8)	0.0007 (8)
C10	0.0149 (9)	0.0156 (10)	0.0148 (10)	−0.0004 (8)	0.0062 (8)	0.0010 (8)
C11	0.0136 (9)	0.0132 (9)	0.0132 (9)	0.0009 (7)	0.0049 (7)	0.0023 (7)
C12	0.0192 (10)	0.0183 (10)	0.0195 (10)	−0.0012 (8)	0.0101 (8)	0.0041 (8)
C13	0.0248 (11)	0.0171 (10)	0.0166 (10)	−0.0012 (8)	0.0092 (8)	0.0060 (8)
C14	0.0207 (10)	0.0139 (10)	0.0143 (10)	0.0014 (8)	0.0055 (8)	0.0035 (8)
C15	0.0152 (9)	0.0139 (9)	0.0119 (9)	0.0018 (7)	0.0040 (7)	0.0030 (7)
C16	0.0257 (11)	0.0178 (10)	0.0148 (10)	0.0056 (9)	0.0041 (8)	0.0073 (8)
C17	0.0201 (10)	0.0207 (11)	0.0158 (10)	0.0069 (8)	0.0018 (8)	0.0061 (8)
C18	0.0148 (9)	0.0186 (10)	0.0153 (10)	0.0034 (8)	0.0034 (8)	0.0033 (8)

### Geometric parameters (Å, °)

**Table:**

Cu1—O6^i^	1.9339 (15)	C4—C5	1.399 (3)
Cu1—O4^ii^	1.9371 (14)	C4—H4	0.9500
Cu1—N2	2.0142 (18)	C5—C6	1.393 (3)
Cu1—N1	2.0166 (17)	C5—H5A	0.9500
Cu1—O1	2.2918 (15)	C6—H6	0.9500
P1—O1	1.5215 (15)	C7—C8	1.406 (3)
P1—O2	1.5341 (16)	C7—H7	0.9500
P1—O3	1.5352 (16)	C8—C9	1.377 (3)
P1—C1	1.805 (2)	C8—H8	0.9500
P2—O6	1.5092 (15)	C9—C10	1.411 (3)
P2—O4	1.5169 (15)	C9—H9	0.9500
P2—O5	1.5741 (15)	C10—C11	1.411 (3)
P2—C3	1.803 (2)	C10—C12	1.435 (3)
O2—H2	0.8400	C11—C15	1.433 (3)
O4—Cu1^iii^	1.9371 (14)	C12—C13	1.360 (3)
O5—H5	0.8400	C12—H12	0.9500
O6—Cu1^i^	1.9340 (15)	C13—C14	1.437 (3)
N1—C7	1.333 (3)	C13—H13	0.9500
N1—C11	1.354 (3)	C14—C15	1.400 (3)
N2—C18	1.333 (3)	C14—C16	1.410 (3)
N2—C15	1.356 (3)	C16—C17	1.376 (3)
C1—C2	1.396 (3)	C16—H16	0.9500
C1—C6	1.401 (3)	C17—C18	1.404 (3)
C2—C3	1.400 (3)	C17—H17	0.9500
C2—H2A	0.9500	C18—H18	0.9500
C3—C4	1.399 (3)		
			
O6^i^—Cu1—O4^ii^	96.46 (6)	C3—C4—H4	120.0
O6^i^—Cu1—N2	160.52 (7)	C6—C5—C4	120.02 (19)
O4^ii^—Cu1—N2	90.51 (7)	C6—C5—H5A	120.0
O6^i^—Cu1—N1	90.87 (7)	C4—C5—H5A	120.0
O4^ii^—Cu1—N1	171.94 (7)	C5—C6—C1	120.71 (19)
N2—Cu1—N1	81.49 (7)	C5—C6—H6	119.6
O6^i^—Cu1—O1	98.12 (6)	C1—C6—H6	119.6
O4^ii^—Cu1—O1	91.46 (6)	N1—C7—C8	122.1 (2)
N2—Cu1—O1	99.88 (6)	N1—C7—H7	118.9
N1—Cu1—O1	90.82 (6)	C8—C7—H7	118.9
O1—P1—O2	111.95 (9)	C9—C8—C7	119.4 (2)
O1—P1—O3	112.39 (9)	C9—C8—H8	120.3
O2—P1—O3	112.01 (9)	C7—C8—H8	120.3
O1—P1—C1	107.19 (9)	C8—C9—C10	119.79 (19)
O2—P1—C1	106.22 (9)	C8—C9—H9	120.1
O3—P1—C1	106.62 (9)	C10—C9—H9	120.1
O6—P2—O4	115.35 (9)	C11—C10—C9	116.6 (2)
O6—P2—O5	110.16 (8)	C11—C10—C12	118.5 (2)
O4—P2—O5	110.15 (8)	C9—C10—C12	124.91 (19)
O6—P2—C3	106.07 (9)	N1—C11—C10	123.45 (19)
O4—P2—C3	109.09 (9)	N1—C11—C15	116.48 (18)
O5—P2—C3	105.48 (9)	C10—C11—C15	120.06 (19)
P1—O1—Cu1	128.96 (9)	C13—C12—C10	121.23 (19)
P1—O2—H2	109.5	C13—C12—H12	119.4
P2—O4—Cu1^iii^	127.78 (9)	C10—C12—H12	119.4
P2—O5—H5	109.5	C12—C13—C14	121.2 (2)
P2—O6—Cu1^i^	138.73 (9)	C12—C13—H13	119.4
C7—N1—C11	118.57 (18)	C14—C13—H13	119.4
C7—N1—Cu1	128.72 (15)	C15—C14—C16	117.0 (2)
C11—N1—Cu1	112.34 (13)	C15—C14—C13	118.7 (2)
C18—N2—C15	118.12 (18)	C16—C14—C13	124.3 (2)
C18—N2—Cu1	128.83 (15)	N2—C15—C14	123.71 (19)
C15—N2—Cu1	112.63 (13)	N2—C15—C11	115.95 (18)
C2—C1—C6	118.83 (18)	C14—C15—C11	120.34 (19)
C2—C1—P1	120.67 (15)	C17—C16—C14	119.2 (2)
C6—C1—P1	120.50 (15)	C17—C16—H16	120.4
C1—C2—C3	121.07 (18)	C14—C16—H16	120.4
C1—C2—H2A	119.5	C16—C17—C18	119.7 (2)
C3—C2—H2A	119.5	C16—C17—H17	120.1
C4—C3—C2	119.43 (18)	C18—C17—H17	120.1
C4—C3—P2	120.71 (15)	N2—C18—C17	122.1 (2)
C2—C3—P2	119.75 (15)	N2—C18—H18	118.9
C5—C4—C3	119.92 (18)	C17—C18—H18	118.9
C5—C4—H4	120.0		
			
O2—P1—O1—Cu1	−2.35 (13)	C2—C3—C4—C5	0.9 (3)
O3—P1—O1—Cu1	124.73 (10)	P2—C3—C4—C5	−175.41 (16)
C1—P1—O1—Cu1	−118.45 (11)	C3—C4—C5—C6	0.4 (3)
O6^i^—Cu1—O1—P1	169.38 (10)	C4—C5—C6—C1	−1.0 (3)
O4^ii^—Cu1—O1—P1	−93.88 (11)	C2—C1—C6—C5	0.4 (3)
N2—Cu1—O1—P1	−3.11 (12)	P1—C1—C6—C5	−178.59 (16)
N1—Cu1—O1—P1	78.39 (11)	C11—N1—C7—C8	0.7 (3)
O6—P2—O4—Cu1^iii^	−106.17 (11)	Cu1—N1—C7—C8	−171.75 (16)
O5—P2—O4—Cu1^iii^	19.28 (13)	N1—C7—C8—C9	−0.1 (3)
C3—P2—O4—Cu1^iii^	134.62 (11)	C7—C8—C9—C10	−0.2 (3)
O4—P2—O6—Cu1^i^	97.16 (15)	C8—C9—C10—C11	−0.1 (3)
O5—P2—O6—Cu1^i^	−28.29 (17)	C8—C9—C10—C12	179.7 (2)
C3—P2—O6—Cu1^i^	−141.97 (14)	C7—N1—C11—C10	−1.0 (3)
O6^i^—Cu1—N1—C7	−16.18 (18)	Cu1—N1—C11—C10	172.65 (16)
N2—Cu1—N1—C7	−178.19 (19)	C7—N1—C11—C15	178.83 (18)
O1—Cu1—N1—C7	81.95 (18)	Cu1—N1—C11—C15	−7.6 (2)
O6^i^—Cu1—N1—C11	171.00 (14)	C9—C10—C11—N1	0.7 (3)
N2—Cu1—N1—C11	9.00 (14)	C12—C10—C11—N1	−179.16 (19)
O1—Cu1—N1—C11	−90.87 (14)	C9—C10—C11—C15	−179.11 (18)
O6^i^—Cu1—N2—C18	110.8 (2)	C12—C10—C11—C15	1.0 (3)
O4^ii^—Cu1—N2—C18	−0.45 (18)	C11—C10—C12—C13	−0.4 (3)
N1—Cu1—N2—C18	178.63 (19)	C9—C10—C12—C13	179.7 (2)
O1—Cu1—N2—C18	−92.02 (18)	C10—C12—C13—C14	−1.1 (3)
O6^i^—Cu1—N2—C15	−76.9 (2)	C12—C13—C14—C15	2.0 (3)
O4^ii^—Cu1—N2—C15	171.83 (14)	C12—C13—C14—C16	−177.0 (2)
N1—Cu1—N2—C15	−9.08 (14)	C18—N2—C15—C14	0.7 (3)
O1—Cu1—N2—C15	80.27 (14)	Cu1—N2—C15—C14	−172.44 (16)
O1—P1—C1—C2	26.20 (19)	C18—N2—C15—C11	−179.11 (18)
O2—P1—C1—C2	−93.64 (17)	Cu1—N2—C15—C11	7.7 (2)
O3—P1—C1—C2	146.75 (16)	C16—C14—C15—N2	−2.1 (3)
O1—P1—C1—C6	−154.87 (16)	C13—C14—C15—N2	178.79 (19)
O2—P1—C1—C6	85.30 (18)	C16—C14—C15—C11	177.76 (19)
O3—P1—C1—C6	−34.31 (19)	C13—C14—C15—C11	−1.4 (3)
C6—C1—C2—C3	0.9 (3)	N1—C11—C15—N2	−0.1 (3)
P1—C1—C2—C3	179.87 (16)	C10—C11—C15—N2	179.72 (18)
C1—C2—C3—C4	−1.5 (3)	N1—C11—C15—C14	−179.95 (18)
C1—C2—C3—P2	174.77 (16)	C10—C11—C15—C14	−0.1 (3)
O6—P2—C3—C4	141.91 (17)	C15—C14—C16—C17	1.3 (3)
O4—P2—C3—C4	−93.26 (18)	C13—C14—C16—C17	−179.6 (2)
O5—P2—C3—C4	25.05 (19)	C14—C16—C17—C18	0.6 (3)
O6—P2—C3—C2	−34.34 (19)	C15—N2—C18—C17	1.3 (3)
O4—P2—C3—C2	90.49 (17)	Cu1—N2—C18—C17	173.27 (16)
O5—P2—C3—C2	−151.21 (16)	C16—C17—C18—N2	−2.0 (3)

Symmetry codes: (i) −*x*, −*y*+2, −*z*+1; (ii) *x*+1, *y*, *z*; (iii) *x*−1, *y*, *z*.

### Hydrogen-bond geometry (Å, °)

**Table:**

*D*—H···*A*	*D*—H	H···*A*	*D*···*A*	*D*—H···*A*
O2—H2···O3^iv^	0.84	1.81	2.489 (2)	136
O5—H5···O1^iii^	0.84	1.74	2.574 (2)	173

Symmetry codes: (iv) −*x*+1, −*y*+2, −*z*+2; (iii) *x*−1, *y*, *z*.
